# Factors related to the risk of illness of nursing staff at work in a psychiatric institution*

**DOI:** 10.1590/1518-8345.3454.3235

**Published:** 2020-02-03

**Authors:** Kayo Henrique Jardel Feitosa Sousa, Regina Célia Gollner Zeitoune, Luciana Fernandes Portela, Gisele Massante Peixoto Tracera, Katerine Gonçalves Moraes, Rachel Ferreira Savary Figueiró

**Affiliations:** 1 Universidade Federal do Rio de Janeiro, Escola de Enfermagem Anna Nery, Rio de Janeiro, RJ, Brazil.; 2 Scholarship holder at the Coordenação de Aperfeiçoamento de Pessoal de Nível Superior (CAPES), Brazil.; 3 Fundação Oswaldo Cruz, Instituto Nacional de Infectologia, Laboratório de Pesquisa Clínica em Doença de Chagas, Rio de Janeiro, RJ, Brazil.; 4 Universidade Federal do Rio de Janeiro, Instituto de Atenção à Saúde São Francisco de Assis, Rio de Janeiro, RJ, Brazil.

**Keywords:** Working Conditions, Psychiatric Nursing, Nursing, Team, Hospitals, Psychiatric, Mental Health, Occupational Health, Condições de Trabalho, Enfermagem Psiquiátrica, Equipe de Enfermagem, Hospitais Psiquiátricos, Saúde Mental, Saúde do Trabalhador, Condiciones de Trabajo, Enfermería Psiquiátrica, Grupo de Enfermería, Hospitales Psiquiátricos, Salud Mental, Salud Laboral

## Abstract

**Objective:**

identify the associations between the sociodemographic, and work variables, health conditions and lifestyles, and the risks of illness of nursing workers in a psychiatric hospital.

**Method:**

analytical cross-sectional study. The sample of 74 workers answered a questionnaire for sociodemographic, work, health conditions and lifestyles survey characterization. The Work Context Assessment Scale and the Human Cost at Work Scale were used to evaluate the perception of the risks of illness in the interviewee’s opinion. A descriptive and bivariate analysis was performed, with significance of 5%.

**Results:**

the factors associated with the risk of illness were: insomnia complaints, night work and workday.

**Conclusion:**

the associations between the work variables, health conditions and life habits can harm the health of the nursing staff of a psychiatric hospital.

## Abstract (pt)

**Objetivo:**

identificar as associações entre as variáveis sociodemográficas, laborais, condições de saúde, hábitos de vida e os riscos de adoecimento do trabalhador de enfermagem de um hospital psiquiátrico.

**Método:**

estudo transversal analítico. A amostra de 74 trabalhadores respondeu a um questionário para caracterização sociodemográfica, laboral, levantamento de condições de saúde e hábitos de vida. Para avaliar a percepção dos riscos de adoecimento, na opinião do entrevistado, foram utilizadas a Escala de Avaliação do Contexto de Trabalho e a Escala de Custo Humano no Trabalho. Realizou-se análise descritiva e bivariada, com significância de 5%.

**Resultados:**

os fatores associados aos riscos de adoecimento foram: queixas de insônia, trabalho noturno e jornada de trabalho.

**Conclusão:**

há evidências de que as associações entre as variáveis laborais, condições de saúde e hábitos de vida podem prejudicar a saúde da equipe de enfermagem de um hospital psiquiátrico.

## Abstract (es)

**Objetivo:**

identificar las asociaciones entre las variables sociodemográficas, laborales, las condiciones de salud, los estilos de vida y de los riesgos de enfermedad de los trabajadores de enfermería en un hospital psiquiátrico.

**Método:**

estudio analítico transversal. La muestra de 74 trabajadores respondió a un cuestionario de caracterización sociodemográfica y laboral, condiciones de salud y estilos de vida. Para evaluar la percepción de los riesgos de enfermedad de acuerdo con el entrevistado, se utilizó la Escala de Evaluación del Contexto Laboral y la Escala de Coste Humano en el Trabajo. Se realizó un análisis descriptivo y bivariado, con una significación del 5%.

**Resultados:**

los factores asociados con el riesgo de enfermarse fueron: las quejas de insomnio, el trabajo nocturno y las horas de trabajo.

**Conclusión:**

existe evidencia de que las asociaciones entre las variables de trabajo, las condiciones de salud y los hábitos de vida pueden perjudicar la salud del personal de enfermería de un hospital psiquiátrico.

## Introduction

The Psychiatric Reform was a milestone in mental health care in Brazil. Since then, many advances can be observed in the care and management of these services, considering the processes of deinstitutionalization and dehospitalization of users, with reorientation of the psychiatric care model^1^. However, areas of care gaps and the need of “replacement of the mental hospitals still operating in Brazil”^2^, in line with the principle of universal coverage of mental health in the country, with emphasis on regions with low economic development.

In this context, mental health services produce situations that affect health conditions, in particular, of nursing professionals. These situations increase the risks of illness associated with the feeling of physical fatigue at the end of the work day, caused by the fear of suffering some kind of aggression by users^3^, exposure to physical and psychic loads, inadequate working conditions, limitation of autonomy, stress complaints, body aches, anxiety and fatigue, leading to suffering at work^4^.

A research^5^ reinforces the occurrence of violence in the context of psychiatry, by identifying a higher prevalence of aggressions by patients to nurses working in psychiatric settings, in addition to presenting a worse subjective state of health and reduced working capacity in relation to those who worked in clinical and surgical units.

Inadequate working conditions and care for people with mental disorders with considerable cognitive demand represent critical risks for the illness of nursing workers^6^^-^ 7. Night work, a common situation among these workers, is a strong factor of occupational exposure, generating important consequences for the work activity and health conditions of the worker^8^^-^ 9. In addition, the results of care - sometimes out of reality -, the state of continuous surveillance and professional dissatisfaction related to environmental structure, comfort, safety and wages should potentiate the possibilities of illness^7^^,^ 10.

The work context requires workers to make use of personal characteristics and physical and mental balance to face the stressful place of work, pressure and responsibilities^11^. Added to this scenario is the exposure to the long working days - commonly observed in nursing teams -, associated with Burnout syndrome, dissatisfaction at work, intention to leave the profession and increased patient dissatisfaction^12^. Thus, the work carried out in a psychiatric institution exposes health professionals to physical and psychological stress, contributing to the development of several diseases and occupational stress.

It is observed, then, that some characteristics of nursing work expose this professional category to different occupational risks and, particularly, worrisome when considering psychiatric institutions. In view of the context of the Brazilian Psychiatric Reform, the problem of professional insecurity regarding its future is still be seen in the hospital care scenario. The *Rede de Atenção Psicossocial* (RAPS - Psychosocial Care Network) foresees deinstitutionalization as a guiding principle and an increase in the number of beds in general hospitals, generating uncertainty among professionals in psychiatric hospitals regarding their insertion in this work process.

Considering the above, the objective of this research was to identify the associations between the sociodemographic, work, health conditions and lifestyle variables and the risks of illness of nursing workers in a psychiatric hospital.

## Method

This is an analytical cross-sectional study, developed in the period from March to April 2016, in a public psychiatric hospital in the Northeast region of Brazil. The hospital - which receives patients from all over the state and several states in the North and Northeast regions - is composed of: Integral Hospitalization Unit with 160 beds divided into male, female, geriatric and clinical pavilions; Anti-Crisis Treatment Unit, with eight beds of attention, four male and four female; Outpatient Service for external care and Emergency Service operating 24 hours a day, with nursing assistance in all the referred units.

The population of this study consisted of 98 nursing professionals working in the psychiatric institution, 18 of them were nurses and 80 nursing technicians/assistants. Of these, five professionals participated in the pre-test and three were on vacation or on leave and the target population was 90 participants (17 nurses and 73 nursing technicians/auxiliaries). The workers who worked in the assistance were considered eligible for the study and those who worked in the administrative area did not provide direct assistance to patients were excluded.

After applying the inclusion and exclusion criteria, the sample was composed of 74 nursing workers, 14 of whom were nurses, 16 nursing assistants and 44 nursing technicians. The final sample of the study represented 82.2% of the target population. The following losses were recorded: eight workers by refusal and eight were not found in the data collection period.

The data collection occurred inside the institution, based on individual interviews, carried out by trained research assistants, five of them undergraduate students in Nursing and one nurse expert in Occupational Nursing. All the assistants received information about the research objectives, methods and techniques, being explained the meanings of the data collection instrument. After the training, the research assistants went to the institution to apply the instrument, programming the schedules for the collection according to the participant’s availability.

The collection instrument consisted of a multithematic questionnaire, structured into two blocks of questions.

The first block included questions related to sociodemographic characterization (sex, age, marital status and children), work (professional category, work hours, shift and number of jobs), health conditions with medical diagnosis and life habits (physical activity, leisure and insomnia complaints).

The second block presented the instruments used to assess the risks of illness at work, namely: the *Escala de Avaliação do Contexto de Trabalho* (EACT) - Work Context Evaluation Scale - composed of 31 items divided into three dimensions - work organization, socio-professional relations and working conditions^13^ and the *Escala de Custo Humano no Trabalho* (ECHT) - Human Cost at Work Scale - which has 32 items divided into the dimensions - emotional cost, cognitive cost and physical cost^13^.

The scales mentioned are part of the *Inventário sobre Trabalho e Risco de Adoecimento* (ITRA) - Inventory about Work and Illness Risk -, a public domain instrument developed in 2003 and validated in 2004 and 2006^13^.

These scales evaluate the risk of illness in the employee’s opinion. To each question is assigned a score ranging from 1 to 5, depending on the intensity of the risk. In other words, the higher the score, the more noticeable the risk, and the final score is obtained by the arithmetic mean of each question, subsequently grouped to form the dimensions of risk of illness. According to the authors of the scales mentioned above, when the score is lower than 2.29, the work environment does not offer risk to the worker; between 2.3 and 3.69, the work environment offers moderate risk; and higher than 3.7, serious risk for worker illness^13^. In this study, in order to maximize the differences between the groups, the classifications were reduced from three to two groups - with risk of illness (score ≥ 2.3) and without risk of illness (score < 2.3).

The data were analyzed with the help of the Statistical Package for the Social Sciences (SPSS) version 21.0. Frequency distribution tables were used to analyze the variables. For bivariate analyses, Pearson’s chi-square test or Fisher’s exact test were used, adopting a statistical significance level of 5%. The estimation of the Odds Ratio (OR) was also evaluated, with a respective confidence interval of 95% (95% CI). The reliability of the scales was evaluated using Cronbach’s alpha coefficient, showing good internal consistency of the set of items that make up the risk factors for illness, ranging from 0.561 to 0.905.

The study was referred to the Research Ethics Committee under CAAE no. 52679216.7.0000.5238, obtaining a favorable report no. 1.434.109. It development complied the ethical precepts of Resolution no. 466/2012 of the National Health Council. Data collection was only initiated after all doubts of the participants were solved and the Informed Consent Form was signed.

## Results

74 (82.2%) workers participated in the study, predominantly female (91.9%), without a partner (54.1%), without children under 6 years of age (87.8%), in the age range between 48 and 72 years (59.5%), with an average of 49 (± 9.22) years. Regarding the characteristics related to work, there was a predominance of professionals who worked up to 30 hours a week (70.3%), with only one job (54.1%) and performed their activities during the night (56.8%). Regarding health conditions and life habits, most participants reported practicing physical activity (56.8%), having time for leisure (78.4%), having at least three health problems diagnosed by a medical professional (74.3%) and not suffering of insomnia (66.2%).

According to the results presented in Table 1, it was possible to observe that insomnia complaints were more frequent among workers at risk of illness for socio-professional relationships. Workers at risk of illness for personal relationships are 4.22 (CI: 1.43-12.41) times more likely to report insomnia complaints. In relation to working hours, we noted its association with both the risk of illness for work organization and working conditions. Although no significant associations were observed with regard to the analysis of the odds ratio, a greater proportion of the risks of illness was observed among workers who work longer than 30 hours per week. 

**Table 1:** Association between the risk of illness, evaluated by the dimensions of work organization, socio-professional relations, working conditions and the sociodemographic, work and related to health and lifestyle variables in nursing workers of a psychiatric hospital, Northeast region of Brazil, 2016 (n = 74)

Variable	n	Illness Risk								
		Work organization			Socio-professional relations			Working conditions		
		n(%)	OR*(95%IC^†^)	p	(n)%	OR*(95%IC^†^)	p	n(%)	OR*(95%IC^†^)	p
Sex^‡^										
Male	06	04(66.7)	1.0		04(66.7)	1.0		06(100)	-	
Female	68	44(64.7)	0.91(0.15-5.37)	0.648	36(52.9)	0.56(0.09-3.27)	0.418	60(88.2)	-	0.490
Age Group										
23 to 47 years old	30	20(66.7)	1.0		19(63.3)	1.0		26(86.7)	1.0	
48 to 72 years old	44	28(63.6)	0.87(0.33-2.32)	0.789	21(47.7)	0.52(0.20-1.36)	0.186	40(90.9)	1.53(0.35-6.69)	0.416
Marital status										
No partner	40	29(72.5)	1.0		21(52.5)	1.0		38(95.0)	1.0	
With partner	34	19(55.9)	0.48(0.18-1.26)	0.136	19(55.9)	1.14(0.45-2.87)	0.771	28(82.4)	0.24(0.04-1.30)	0.085
Children (≤ 6 years)										
No	65	43(66.2)	1.0		34(52.3)	1.0		59(90.8)	1,0	
Yes	09	05(55.6)	0.64(0.15-2.62)	0.391	06(66.7)	1.82(0.42-7.92)	0.329	07(77.8)	0.35(0.06-2.11)	0.249
Category										
Nurse	14	09(64.3)	1.0		06(42.9)	1.0		11(78.6)	1.0	
Assistant/technician	60	39(65.0)	1.03(0.30-3.47)	0.595	34(56.7)	1.74(0.53-5.64)	0.351	55(91.7)	3.00(0.62-14.43)	0.169
**Work hours** ^‡^										
≤ 30 hours per week	52	30(57.7)	1.0		26(50.0)	1.0		44(84.6)	-	
> 30 hours per week	22	18(81.8)	3.30(0.97-11.12)	0.047	14(63.6)	1.75(0.62-4.87)	0.282	22(100)	-	0.051
Number of jobs										
Just one	40	27(67.5)	1.0		24(60.0)	1.0		38(95.0)	1.0	
Two or more	34	21(61.8)	0.77(0.29-2.02)	0.607	16(47.1)	0.59(0.23-1.49)	0.266	28(82.4)	0.24(0.04-1.30)	0.085
Night shift										
No	32	20(62.5)	1.0		14(43.8)	1.0		28(87.5)	1.0	
Yes	42	28(66.7)	1.20(0.45-3.13)	0.710	26(61.9)	2.08(0.82-5.32)	0.121	38(90.5)	1.35(0.31-5.89)	0.683
Physical activity										
Yes	42	29(66.7)	1.0		23(54.8)	1.0		36(85.7)	1.0	
No	32	20(62.5)	0.83(0.31-2.17)	0.710	17(53.1)	0.93(0.37-2.35)	0.889	30(93.8)	2.50(0.47-13.30)	0.238
Leisure time										
Yes	58	36(62.1)	1.0		31(53.4)	1.0		51(87.9)	1.0	
No	16	12(75.0)	1.83(0.52-6.39)	0.337	09(56.3)	1.12(0.36-3.41)	0.842	15(93.8)	2.05(0.23-18.08)	0.507
Health problems										
Three diagnostics	55	36(65.5)	1.0		30(54.5)	1.0		49(89.1)	1.0	
≥ 4 diagnostics	19	12(63.2)	0.90(0.30-2.67)	0.857	10(52.6)	0.92(0.32-2.63)	0.885	17(89.5)	1.04(0.19-5.65)	0.666
Insomnia complaints										
No	49	30(61.2)	1.0		21(42.9)	1.0		43(87.9)	1.0	
Yes	25	18(72.0)	1.62(0.57-4.63)	0.358	19(76.0)	4.22(1.43-12.41)	0.007	23(92.0)	1.60(0.30-8.59)	0.451

* OR = Odds Ratio;

† CI = Confidence interval;

‡ not possible to calculate the measure of association between the indicated variable and working conditions

The data in Table 2 show that the relationship between the risk of illness and the study variables was expressed in the significant association between physical cost and insomnia complaints. Individuals who reported insomnia complaints are approximately three times more likely to present risk of illness for physical cost at work. A similar result is observed when evaluating the night shift, which is 2.70 (CI: 1.05-6.99) times higher in those with risk of illness.

**Table 2:** Association between the risk of illness, evaluated by the dimensions affective cost, cognitive cost, physical cost and the sociodemographic, labor, and health-related variables and life habits in nursing workers of a psychiatric hospital, Northeast region of Brazil, 2016 (n = 74)

Variable	n	Risk of illness								
		Affective cost			Cognitive cost			Physical cost		
		n(%)	OR*(95%IC^†^)	p	(n)%	OR*(95%IC^†^)	p	n(%)	OR*(95%IC^†^)	p
Sex										
Male	06	03(50.0)	1.0		05(83.3)	1.0		02(33.3)	1.0	
Female	68	32(47.1)	0.88(0.16-4.72)	0.609	54(79.4)	0.77(0.08-7.14)	0.649	36(52.9)	2.25(0.38-13.11)	0.311
Age Group										
23 to 47 years old	30	13(43.3)	1.0		24(80.0)	1.0		15(50.0)	1.0	
48 to 72 years old	44	22(50.0)	1.30(0.51-3.32)	0.573	35(79.5)	0.97(0.30-3.09)	0.962	23(52.3)	1.09(0.43-2.77)	0.848
Marital status										
No partner	40	17(42.5)	1.0		30(75.0)	1.0		18(45.0)	1.0	
With partner	34	18(52.9)	1.52(0.60-3.81)	0.370	29(85.3)	1.93(0.58-6.34)	0.272	20(58.8)	1.74(0.69-4.40)	0.236
Children (≤ 6 years)										
No	65	30(46.2)	1.0		51(78.5)	1.0		35(53.8)	1.0	
Yes	09	05(55.6)	1.45(0.35-5.92)	0.430	08(88.9)	2.19(0.25-19.06)	0.415	03(33.3)	0.42(0.09-1.86)	0.213
Category										
Nurse	14	06(42.9)	1.0		11(78.6)	1.0		05(35.7)	1.0	
Assistant/technician	60	29(48.3)	1.24(0.38-4.03)	0.712	48(80.0)	1.09(0.26-4.53)	0.580	33(55.0)	2.20(0.65-7.34)	0.158
Work hours										
≤ 30 hours per week	52	22(42.3)	1.0		40(76.9)	1.0		23(44.2)	1.0	
> 30 hours per week	22	13(59.1)	1.97(0.71-5.42)	0.186	19(86.4)	1.90(0.47-7.53)	0.278	15(68.2)	2.70(0.94-7.72)	0.060
Number of jobs										
Just one	40	22(55.0)	1.0		30(75.0)	1.0		21(52.5)	1.0	
Two or more	34	13(38.2)	0.50(0.20-1.28)	0.150	29(85.3)	1.93(0.58-6.34)	0.272	17(50.0)	0.90(0.36-2.25)	0.830
Night shift										
No	32	13(40.6)	1.0		24(75.0)	1.0		12(37.5)	1.0	
Yes	42	22(52.4)	1.60(0.63-4.07)	0.316	35(83.3)	1.66(0.53-5.20)	0.377	26(61.9)	2.70(1.05-6.99)	0.037
Physical activity										
Yes	42	19(45.2)	1.0		35(83.3)	1.0		22(52.4)	1.0	
No	23	16(50.0)	1.21(0.48-3.04)	0.684	24(75.0)	0.60(0.19-1.87)	0.377	16(50.0)	0.90(0.36-2.28)	0.839
Leisure time										
Yes	58	27(46.6)	1.0		44(75.9)	1.0		27(46.6)	1.0	
No	16	08(50.0)	1.14(0.37-3.47)	0.807	15(93.8)	4.77(0.57-39.43)	0.105	11(68.8)	2.52(0.77-8.19)	0.116
Health problems										
Three diagnostics	55	26(47.3)	1.0		42(76.4)	1.0		28(50.9)	1.0	
≥ 4 diagnostics	19	09(47.4)	1.00(0.35-2.85)	0.994	17(89.5)	2.63(0.53-12.92)	0.188	10(52.6)	1.07(0.37-3.04)	0.555
Insomnia complaints										
No	49	21(42.9)	1.0		36(73.5)	1.0		21(42.9)	1.0	
Yes	25	14(56.0)	1.69(0.64-4.48)	0.284	23(92.0)	4.15(0.85-20.12)	0.061	17(68.0)	2.83(1.02-7.80)	0.041

* OR = Odds Ratio;

† IC = Confidence interval

## Discussion

The sociodemographic characteristics of the sample are similar to those found in other studies^14^^-^ 15, reinforcing the profile of the Brazilian nursing workforce with a predominance of female and adult-young professionals.

The participants in this study were in risk of illness fundamentally associated with the work context. Our findings confirm the vulnerability of the nursing staff to illness due to stressors in the work environment, such as physical and psychological demands, repetition of tasks, pressures and responsibilities, need for constant attention, ergonomic risks, manipulation of materials with risk of exposure to contaminated fluids, wage dissatisfaction and non-recognition by peers^14^.

In this study, referrals to insomnia are associated with the risk of illness both for socio-professional relationships and for physical costs. In this regard, a study proved that the nursing staff of psychiatric hospitals recognizes more the risks to their physical health than to the psychic, denoting an arduous and dangerous work. It also reinforced the need to expand the scope of knowledge of workers about what constitutes risk to their health, considering that less than half of the study sample communicated its last exposure to some occupational risk to the management bodies of hospitals^16^.

Previous studies demonstrated that sleep disorders were reported by workers with Burnout^17^, even though there was an association between insomnia and musculoskeletal disorders - such as headaches, back and neck pain^18^ - and that the number of nursing professionals who attend work complaining of some musculoskeletal symptom is considerable^19^. Some studies^14^^,^ 18 ^-^ 20 have indicated that the main body sites of musculoskeletal disorders are the regions of the head and cervical, lumbar and thoracic spine.

Thus, insomnia associated with the risk of illness for physical cost represents an occupational health problem that usually has multifactorial causality, including the physical, psychosocial and ergonomic dimensions.

In addition, it is noteworthy that sleep dissatisfaction was associated with patterns of negative and sometimes “cynical” attitudes and feelings, reinforcing the findings of this research^21^. In this line, it is important to point out that social support, professional recognition by patients, superiors and other team members, optimism, pleasure and job satisfaction are protective factors for not getting sick and cordiality and respect relationships should be strengthened^22^.

The study also measured the association between night work and the dimensions of risk of illness, and it was found that it is approximately three times more likely to present risk of illness for physical cost among night shift workers than those who works during the day. Literature data corroborate the findings of this investigation, identifying that working at night increases the risk of depersonalization^22^, with higher prevalence of mood swings and headaches among night workers^23^. After night work, the risk of headaches and upper limbs increased; when associated with a few hours of sleep, it also increases the risk of abdominal and back pain.^24^.

Despite having lower labor demands and higher financial gains - considering the additional wages in relation to daytime workers -, nursing professionals in the night shift have a fragmented work process with interpersonal relationships often conflicting and/or absent, due to low supervision and lower staff numbers, which can cause social isolation and physical and mental illness^21^.

Thus, the importance of the balance between private and professional life is outlined and also the importance of avoiding dissonances between the chronotype and the work shift, thus minimizing biological disorders, the commitment of professional performance and social and family relationships^25^.

Given the findings presented here - greater chances of risk of illness related to physical cost among those workers who work at night and reported complaints of insomnia -, it is pointed out the possibility of the amount of hours of sleep act as a mediator between the work shift and the risk of illness, corroborating a previous study^24^.

Although this investigation did not find a significant difference in the odds ratio between the different working days, the study showed higher prevalence of illness among workers with long working hours^26^. The illness resulting from long working days can be explained by the reduction of free time - for sleep and recovery, family and social experience in addition to the longer exposure time to the risks of illness limited to the work environment^27^. This aspect was reported by nurses in qualitative research, when they attributed the illness to work overload and lack of time for self-care^28^.

Nursing still appears as the professional category that performs the longest working day among health care professionals^29^. A study revealed that, despite positively understanding their work as a care relationship, nursing workers recognize the deficiencies and deficits associated with the organization and working conditions - such as work overload, lack of autonomy and deficit of staff and material -, thus pointing to an ambivalent work^30^. It is important to highlight that long working days were associated with inadequate working conditions, work overload and stress^31^.

A study pointed out that the reduction of the workday makes the work more pleasant, with improved recovery, lower energy requirements, improvements in the quality of care and promotion of balance between personal and professional life^32^. These results are consistent with the current debates in favor of reducing the weekly working hours, considering that this factor is characterized as a systemic stressor, representing a serious and challenging occupational health problem.

Although there was no significant reference - in this research - to the impact on the psychic sphere (affective and cognitive cost) of the individuals interviewed, the work within psychiatric hospitals has great potential to affect their emotional stability.

In addition to acute situations or psychotic outbreaks, which place all workers in psychiatric institutions in a constant state of alert and emotional tension, patients in a psychiatric institution are individuals stigmatized by society and are in the process of rehabilitation. The nursing team, being the one that most have contact with these individuals - being, therefore, more time in a face to face relationship with the suffering of patients -, deals with chronic events, whose horizon or perspective of improvement or cure is slow, perhaps, distant. This reality may translate into frustration for the nursing team, which usually does not see the return of their effort and commitment.

Working in an environment without the proper structure, without the recognition and appreciation of colleagues who share the work environment, together with low wages and long working hours, are also factors that negatively affect the health of these workers, making them more vulnerable to illness. On the other hand, factors such as social recognition, good work relations, good pay and fair working hours act as protective elements should be reinforced to avoid illness.

The strengthening of the set of knowledge of nursing workers about what may constitute a real risk to their health, using in-service education actions, should also be considered. Once clarified about the different kinds of risks to which they are exposed in their work environment, this nursing worker can create strategies to minimize the different risks to their health, not only physically, but also emotionally.

The results of this study, however, alert us to think about solutions for night shift workers that are usually outside the scope of in-service education initiatives. In other words, educational actions are generally designed and implemented for day shift workers. This “isolation/distancing” from the other members of the nursing team can lead to a feeling of “not being part” of the group, further removing the night shift worker from the feeling of being part of a team that works collaboratively aimed at the recovery of the individual with mental health problems.

The limitations of the study are due to the size of the sample and its design, because, despite meeting the proposed objectives, 74 nursing professionals in the hospital area do not standardize the nursing context in psychiatry existing in Brazil, and make it impossible to perform more elaborate statistical analyses, besides not being possible to make causal inferences.

## Conclusion

The results confirmed associations between insomnia complaints, night work and working days of more than 30 hours per week and the risks of illness, mainly associated with the work context. Thus, we concluded that there is evidence that the associations between the work variables, health conditions and life habits and the risks of illness related to the dimensions of the context and human cost at work can harm the health of the nursing staff working in a psychiatric hospital. Thus, this study will greatly contribute to the knowledge of the risk factors that generate illness of professionals and more specifically the intensity and perception of these factors by professionals, in the context of work in the psychiatric nursing area.
